# Hypoxia-related biological markers as predictors of epirubicin-based treatment responsiveness and resistance in locally advanced breast cancer

**DOI:** 10.18632/oncotarget.20239

**Published:** 2017-08-14

**Authors:** Manuela Milani, Sergio Venturini, Simone Bonardi, Giovanni Allevi, Carla Strina, Maria Rosa Cappelletti, Silvia Paola Corona, Sergio Aguggini, Alberto Bottini, Alfredo Berruti, Adrian Jubb, Leticia Campo, Adrian L. Harris, Kevin Gatter, Stephen B. Fox, Daniele Generali, Giandomenico Roviello

**Affiliations:** ^1^ U.O. Multidisciplinare di Patologia Mammaria, U.S Terapia Molecolare e Farmacogenomica, ASST Cremona, Viale Concordia 1, Cremona, Italy; ^2^ CE.R.G.A.S., Università Bocconi, Milano, Italy; ^3^ Peter MacCallum Cancer Centre, Bentleigh East VIC, Australia; ^4^ U.O. Oncologia Medica, Spedali Civili si Brescia, University of Brescia, Brescia, Italy; ^5^ Molecular Oncology Laboratories, Weatherall Institute of Molecular Medicine, University of Oxford, John Radcliffe Hospital, Oxford, OX3 9DS, UK; ^6^ Peter MacCallum Cancer Centre, St Andrews Place, East Melbourne, Victoria, Australia; ^7^ Department of Medical, Surgery and Health Sciences, University of Trieste, Piazza Ospitale 1, Trieste, Italy; ^8^ Department of Oncology, Medical Oncology Unit, San Donato Hospital, Italy

**Keywords:** epirubicin resistance, haemoglobin, hypoxia-inducible factor, neoadjuvant, breast cancer

## Abstract

**Purpose:**

To identify hypoxia-related biomarkers indicative of response and resistance to epirubicin treatment in patients with locally advanced breast cancer.

**Patients and Methods:**

One hundred seventy-six women with T2-4 N0-1 breast tumours were randomly assigned to receive epirubicin 120 mg/m2/1-21 (EPI ARM), epirubicin 120 mg/m2/1-21 + erythropoietin 10.000 IU sc three times weekly (EPI-EPO ARM) and epirubicin 40 mg/m2/w-q21 (EPI-W ARM). Sixteen tumour proteins involved in cell survival, hypoxia, angiogenesis and growth factor, were assessed by immunohistochemistry in pre-treatment samples. A multivariate generalized linear regression approach was applied using a penalized least-square minimization to perform variable selection and regularization.

**Results:**

VEGF and GLUT-1 expression were significantly positively associated with complete response (CR) to treatment in all leave-one-out iterations. Bcl-2 expression was inversely correlated with pCR, whilst EPO expression was positively correlated with pathological complete response (pCR). Haemaglobin and HIF-1a nuclear expression were inversely correlated with pCR. HB and HIF-1a expression were associated with a higher risk of relapse and overall survival.

**Conclusion:**

Hypoxic biomarkers determines the epirubicin resistance in breast cancer. Assessment of such biomarkers, may be useful for predicting chemosensitivity and also anthracycline-based treatment outcome.

## INTRODUCTION

Current chemotherapy treatment practice applies therapy selection decisions empirically despite the observation that all regimens are not equally effective across patients. Thus specific predictors of response for this therapy are urgently required to select appropriate patients with breast cancer, provide clear directions for clinicians, and improve patients’ cancer journey [1].

It is recognised that anthracyclines reduce blood flow [2] leading to induction of tumour hypoxia-related growth factors [3] such as vascular endothelial growth factor (VEGF), which paradoxically antagonise anthracylin’s therapeutic effect [4]. In this context, we previously showed that low haemoglobin (Hb) levels affect the efficacy of antineoplastic agents in patients with breast cancer, the corollary being a potential benefit of co-administration of erythropoietin to maintain oxygen delivery to the tumour bed [5], modulate tumour angiogenesis and thereby enhance the therapy effectiveness [6]. To test this effect. we initiated a randomised neoadjuvant clinical trial comparing two different schedules of neo-adjuvant epirubicin (EPI) single treatment, a “1-21 schedule” versus “weekly schedule” plus or minus the concomitant administration of EPO in patients with locally advanced breast cancer.

The aims of this study was 1) to assess whether the addition of EPO improves clinical or pathology response 2) identify whether hypoxia-related biomarkers can identify resistance or response to EPI±EPI and 3) evaluate whether such biomarkers provides prognostic information for disease free or overall survival.

## RESULTS

### Clinical response to treatment

One hundred and seventy six patients were evaluated for disease response; three patients were not evaluated due to discontinuation of treatment. One hundred and sixty-three of 176 patients (92.6%) showed a clinical response (CR or PR): 57/63 (90.5%) in the EPI 120 arm, 54/55 (98.2%) in the EPI 120 + EPO arm and 52/58 (89.7%) in the EPI 40+EPO arm, respectively. Complete Response (CR) was obtained in 60 patients (34.1%), 16 (25.4%) in EPI 120 arm, 18 (32.7%) in EPI 120 + EPO arm and 26 (44.8%) in EPI 40 + EPO arm, respectively. We compared responders with non-responders (NR) (i.e. PR plus CR, 57 [90.5%] *vs.* SD plus Progression, 6 [9.5%] for the EPI 120 group) with the aim of identifying markers involved in response to EPI. Furthermore, to determine markers associated with sensitivity to EPI, we compared the CR, 60 patients (34.1%) versus PR or NR, 116 patients [65.1%] (Supplementary Table 1).

**Figure 1:**
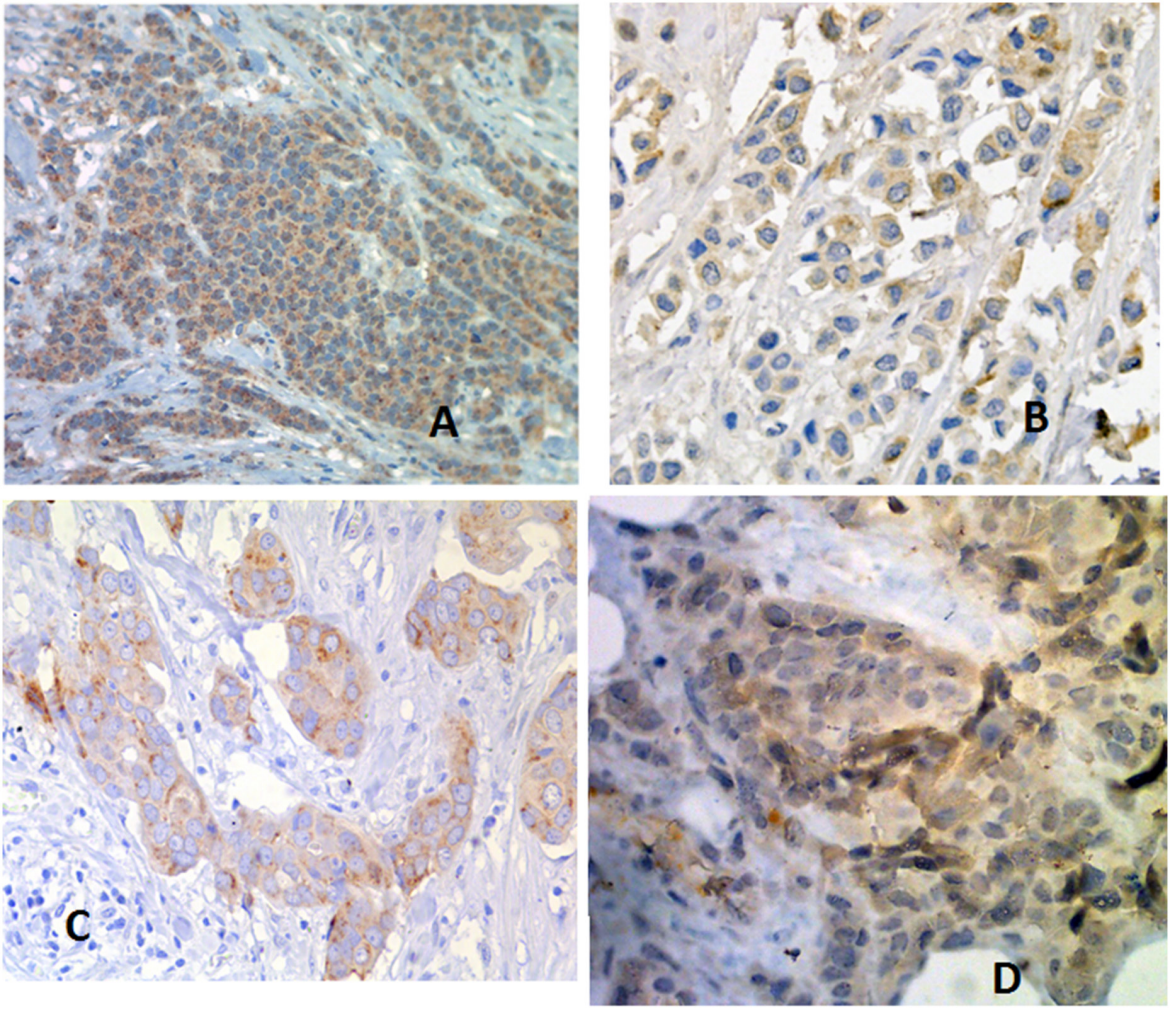
Immunohistochemistry of most significant factors associated with response to neo-adjuvant treatment **(A)** EPO; **(B)** VEGF; **(C)** GLUT-1; **(D)** HIF-1? omit.

Immunohistochemistry (Figure 1) is shown for most significant factors associated with response to neo-adjuvant treatment including EPO; VEGF; GLUT-1 and HIF-1.

### Factors associated with epirubicin sensitivity (CR)

**Figure 2:**
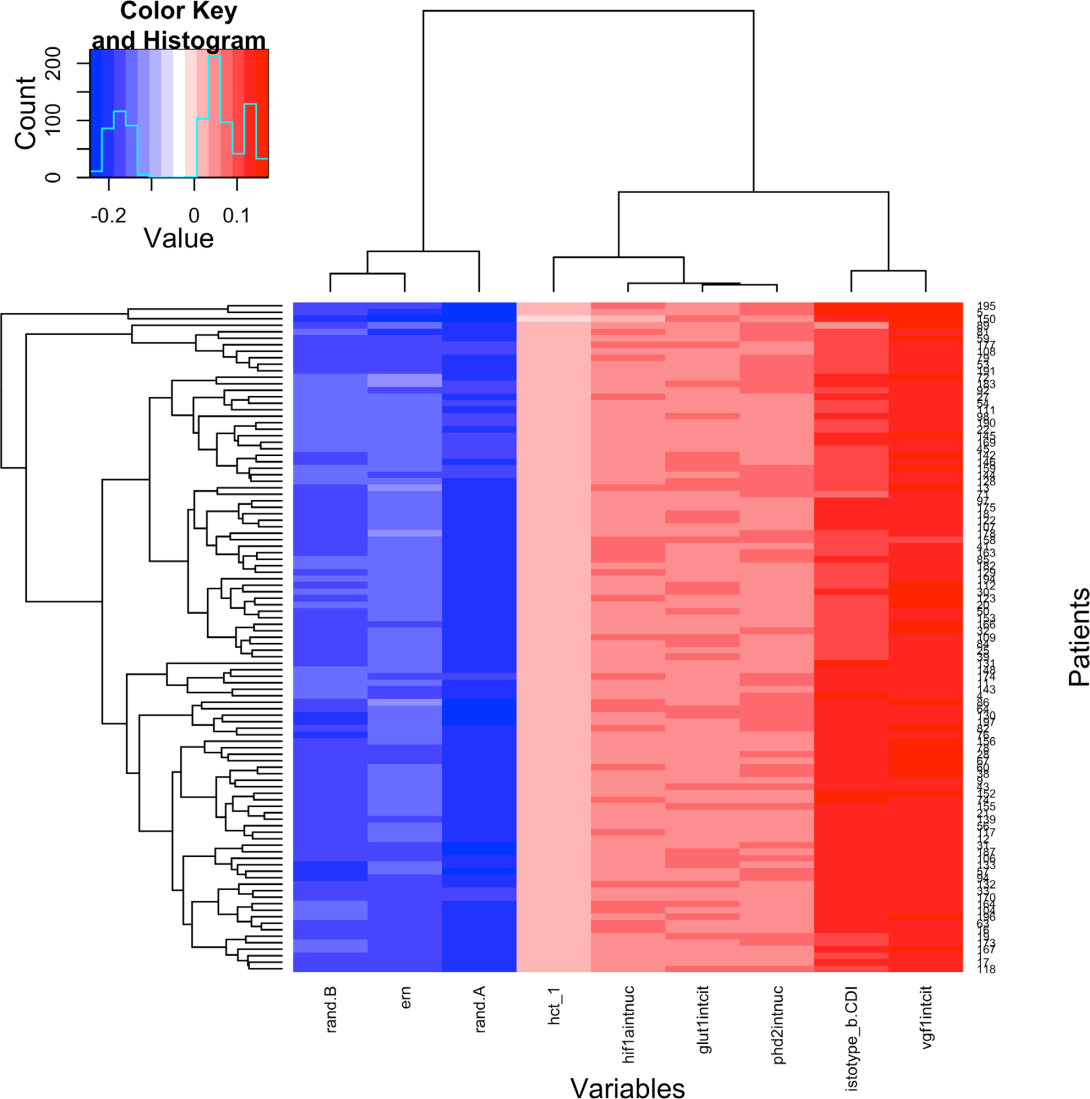
Factors Associated With Epirubicin Sensitivity

The elastic-net reduced model for sensitivity showed that nuclear expression of VEGF was positively correlated with sensitivity to EPI in all leave-one-out iterations, and cytoplasmic expression of GLUT-1 was positively correlated with CR in 94 out of 108 (87%) iterations (Figure 2). GLUT-1 expression estimated coefficients showed slightly smaller values compared with those of VEGF expression. ER expression was inversely correlated with complete response (CR) in 71 of the 108 fits (≈66%). None of these factors showed significant interaction with treatment in any of the leave-one-out iterations. All other variables were significant in less than 40% of the leave-one-out iterations or not significant. The test of the model on the cases, which were left out from the model building (one at each iteration) gave an area under the ROC curve of 53% with a 95% confidence interval of 41% to 65%; thus the null hypothesis of area under the curve [7] equal to 50% (random prediction) could not be rejected.

### Factors associated with response to epirubicin (non-responders vs responders)

**Figure 3:**
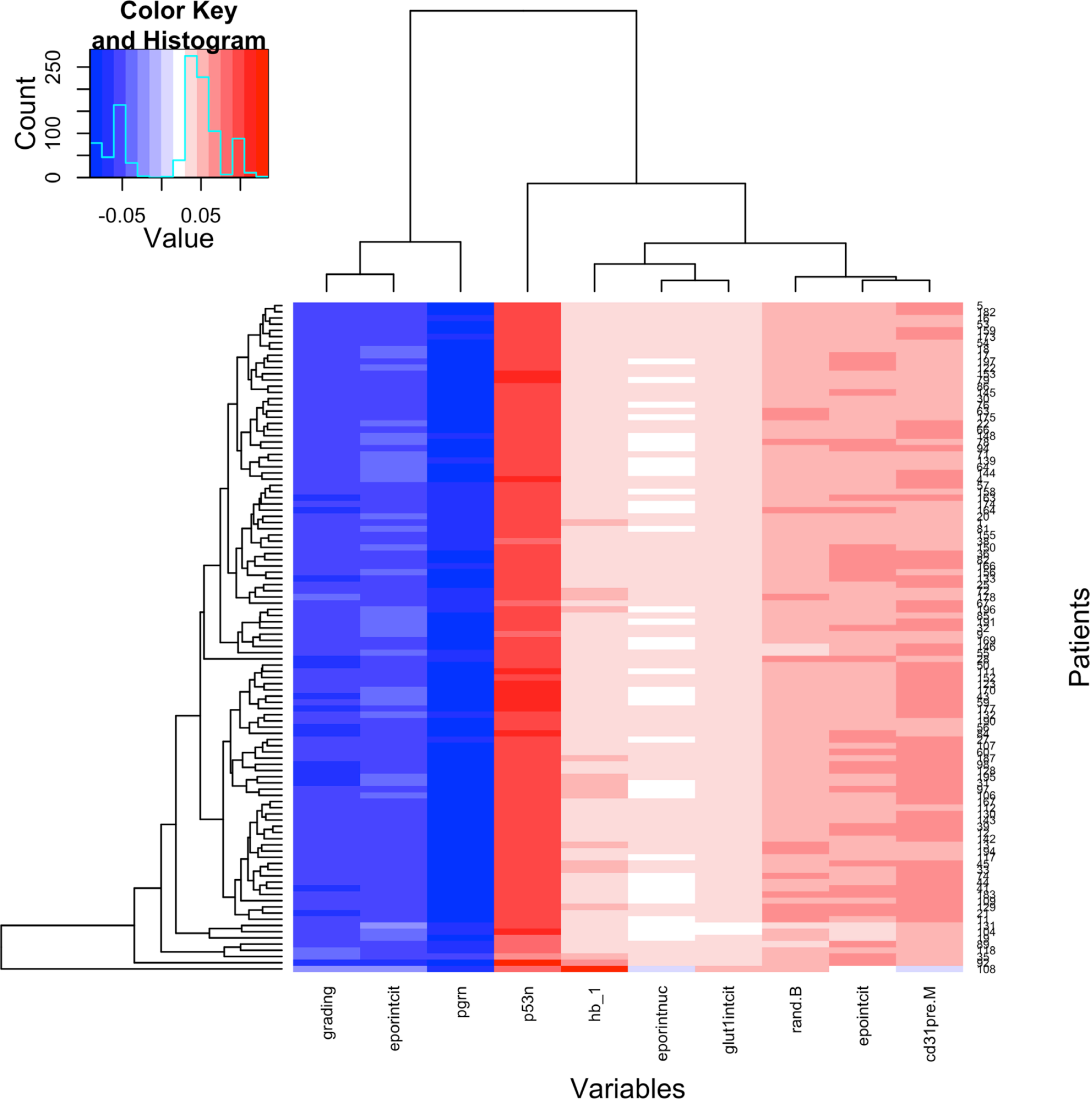
Factors Associated With Epirubicin Response

PgR nuclear expression intensity and basal haemoglobin (Hb) level was the only significant prognostic factor in the reduced model of EPI resistance in all leave-one-out iterations. PgR consistently showed an inverse association with either partial or complete response, while Hb levels were positively correlated with treatment response (Figure 3). GLUT-1 expression was also positively associated with response to treatment in all leave-one-out iterations and p53 expression showed a significant positive correlation with clinical response in 103 of the 108 fits (≈95%). All other markers scores were significant in less than 50% of the leave-one-out iterations or not significant. Neither of these factors showed interaction with treatment in any of the leave-one-out iterations. The test of the model on the cases which were left out from the model building (one per iteration) gave an area under the ROC curve of 60% with a 95% confidence interval of 41% to 81% so that the null hypothesis of AUC equal to 50% (random prediction) could not be rejected.

### Factors associated with pathological complete response (pCR)

**Figure 4:**
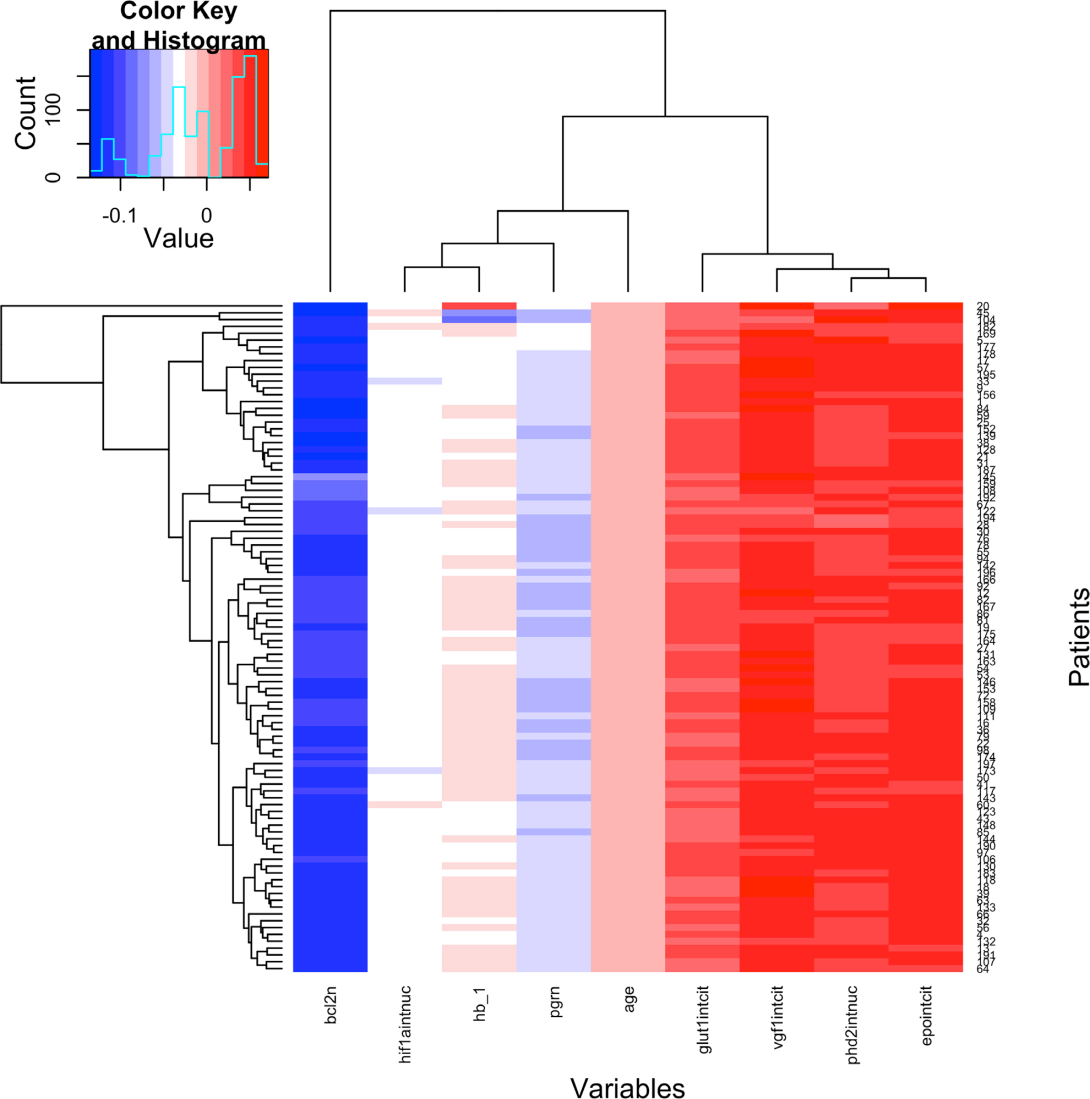
Factors Associated With Pathological Complete Response

The reduced elastic net model for pathological complete response showed that bcl-2 expression inversely correlated with pathological complete response (≈99%), whilst cytoplasmic EPO expression intensity showed direct correlation with pathological complete response in 98 out of 99 (≈99%) leave-one-out iterations (Figure 4).

Hb basal levels and nuclear HIF-1α expression intensity were both inversely correlated with pCR in 56 of the 99 fits (≈57%). All the other variables were significant in <50% of the leave-one-out iterations or not significant. The test of the model on the cases, which were left out from the model building (one at each iteration) gave an area under the ROC curve of 58% with a 95% confidence interval of 41% to 75%; thus the null hypothesis of area under the curve [7] equal to 50% (random prediction) could not be rejected.

### Factors associated with disease free survival (DFS) and overall survival (OS)

The reduced elastic net model for DFS showed that Hb basal level was associated with a higher risk of relapse in 97 of 108 (≈90%) leave-one-out iterations (Supplementary Figure 1). Additionally, nuclear HIF-1 α expression also showed a positive correlation with a higher risk of relapse in 64 of 108 (≈59%) iterations.

The reduced elastic net model for OS showed that Hb basal level was associated with a higher risk of death in 98 of 108 (≈91%) leave-one-out iterations. HIF-1 α expression intensity was also correlated with the risk of death in 84 out of 108 (≈78%) iterations (Supplementary Figure 2). All other variables were significant in less than 50% of the leave-one-out iterations or not significant.

## DISCUSSION

The aim of neo-adjuvant therapy in breast cancer is to reduce the size of the primary tumour, thereby allowing for a conservative surgical approach [8]. Also, by allowing the possibility of “taking a second look”, evaluating the pathological response to therapy “*in vivo*”, a neoadjuvant approach helps in the process of identifying the post-operative treatment strategies most suitable for a given patient and likely to succeed [9]. For these reasons, the neo-adjuvant approach represents the ideal scenario to allow treatment personalization on the basis of specific features of the tumour at diagnosis and after surgery. Several randomized studies have shown that anthracyclines, in association with taxanes, provide high rates of pCR in the neo-adjuvant setting [10, 11]; however, this therapeutic approach is ineffective in approximately half of the patients and associated with the risk of serious side effects [12]. The identification of reliable predictive factors would help clinicians with the selection of the most appropriate therapy for individual patients. In this context, it has been demonstrated that different breast cancer molecular sub-groups have differing pCR and survival rates. This is true in particular with regard to triple negative and HER-2-enriched breast cancer subgroups, whereas such a correlation is still controversial for the luminal subtypes [13]. Nevertheless, although H*ER-2* and *topoisomerase II alpha* (*TOP2A)* immunohistochemical expression have been proposed as markers of sensitivity to anthracyclines some studies suggest that these two combined markers do not necessarily identify the only patients that will benefit from anthracyclines [14]. Thus, to date, no specific biomarkers have been identified that are able to predict tumour response to anthracycline-based chemotherapy.

Tumour hypoxia has been traditionally considered an obstacle to overcome in the treatment of solid tumours as it is associated with resistance to radiation and chemotherapy [15]. Our group previously showed that high expression of VEGF, GLUT-1, p53, markers of angiogenesis [16], tumour metabolism [17] and tumour proliferation [18, 19], and low expression of ER, are predictors of response to epirubicin. Among these, the role of GLUT-1 is still controversial, in fact it is unknown whether GLUT-1 is associated to hypoxia in breast cancer cells or it is a hypoxia-independent feature of transformed cells which display altered metabolism, as in other cancers [20]. Therefore, the discrepancy between the expression of hypoxia-markers and the rate of response to anthracyclines/DFS/OS that we found in this study could be explained by the fact that GLUT-1 is perhaps expressed in breast cells irrespective of the degree of hypoxia, or independently from it. A recent report demonstrated a requirement for GLUT-1 in mammary tumourigenesis [20, 21] as a consequence of the fact that early malignant transformation requires glucose. The metabolic alterations typically found in tumour cells, more than the response to hypoxia, could explain our results. Taken together our results may open the way to studies which could investigate inhibitors of GLUT-1 in combination with anthracyclines. It is well known that low expression of ER is related to an increased Ki67 index [22, 23], as well as amplification of HER-1/HER-2 signalling [24], increased tumour-angiogenesis [16] and metabolism [17]. Moreover high expression of p53, as detected by immunohistochemistry [25], is associated with inhibition of tumour apoptosis resulting in highly proliferative tumours, more likely to respond to chemotherapy [8, 26]. On the other hand, we found that high expression of Pg receptors correlates with anthracycline treatment resistance. This fact could further strengthen the evidence of an existing relation between increased expression of steroid hormone receptors and aggressiveness of disease in breast cancer [27].

Further to this, we found that high HB levels and immunohistochemistry expression of bcl-2 and HIF-1α were inversely correlated with pCR. In 2012, Tanaka et al demonstrated that anthracycline chemotherapeutic agents inhibit binding of the HIF-α/ARNT heterodimer to the target gene enhancer and reduce response to hypoxia [28]. Considering that GLUT-1 expression is controlled by HIF-1 and decreased oxidative phosphorylation [29], it may be that anthracyclines act by uncoupling the two mechanisms and that doxorubicin and daunorubicin act as potent inhibitors of HIF-1α -mediated gene transcription [30]. From our work, we can confirm the role of HIF-α expression as a marker of response during anthracycline-based chemotherapy and therefore hypothesize the possible use of anti-HIF-α in treatment of breast cancer.

Importantly, we found that EPO expression in the tumour context correlated with pCR. Hypoxia is associated with the induction of EPO and EPOR mRNA expression along with their related proteins in breast carcinomas [31]. EPO expression in turn stimulates tyrosine phosphorylation, DNA synthesis, migration of vascular endothelial cells, angiogenesis and proliferation in breast cancer cells [32, 33]. Furthermore, EPO also leads to increased expression of anti-apoptotic proteins such as bcl-2 and bcl-XL [34, 35] and their activation is likely to account, at least in part, for the enhanced anti-tumour effect of anthracycline-based chemotherapy which accompanies a pCR.

Basal HB levels (≥ 13 gr/dl-1) expression was positively correlated with pCR as previously observed [5]. Haemoglobin blood concentration is responsible for the availability of oxygen within the tumour; high HB and therefore high oxygen delivery may cause the generation of hydrogen peroxide or nitric oxide, responsible of oxidative damage to tumour cells and induction of apoptosis [36]. However, oxygen distribution in the context of the tumour is heterogeneous, with the presence of very “low-oxygen” areas, predominantly because of severe structural abnormalities of the tumour microvessels, disturbed microcirculation, and tumour-related anaemia [37].

In conclusion, predicting response to anthracycline-based therapy is an ongoing challenge in breast cancer patients’ management. Immunohistochemistry expression of hypoxia-related markers could help identifying patients who will benefit from this type of chemotherapy. Co-administration of hypoxia-targeting agents could provide further benefit. Moreover, routine haemoglobin levels testing during treatment, and correction of anaemia with concomitant administration of erythropoietin, could be of significant importance as it seems to directly affect patients’ outcome.

Finding biological markers of response to systemic and/or targeted therapies represents an inevitable step towards “personalised” medicine.

## MATERIALS AND METHODS

### Patients and treatment evaluation

This was a single center, randomized, phase II trial which enrolled 198 patients with T2-4 N0-1 primary breast cancer, from January 2002 to November 2005. The trial was constituted of four treatment arms: Epirubicin 120 mg/m^2^/1-21 (66 patients) (EPI ARM), Epirubicin ARM 120 mg/m^2^/1-21 + erythropoietin (epoetin) alfa 10.000 IU sc three times weekly (57 patients) (EPI-EPO ARM), Epirubicin 40 mg/m^2^/w-q21 (62 patients) (EPI-WARM) and Epirubicin 40 mg/m^2^/w-q21+ erythropoietin (epoetin) alfa 10.000 IU three times weekly (13 patients) (EPI-EPO-W ARM). In the EPI-EPO arm erythropoietin was systematically administered when haemoglobin dropped below 12.0 g/dl during treatment. The authors originally planned to administer four cycles in all treatment arms but the EPI-EPO-W ARM was stopped early (and not included in the analysis) due to the high rate of patients’ refusal to self-administer erythropoietin (epoetin) alfa. Thus, the patients originally assigned to this arm were randomly reallocated to the other arms. In order to avoid bias, it was decided to exclude the 13 patients allocated in the EPI-EPO-W ARM from the analysis, therefore decreasing the sample size to 185 patients in total. In 9 patients, the response indicator was unavailable for technical reasons and thus these patients were also excluded from the analysis. Hence, the final sample-size was composed of 176 patients. Patients’ characteristics are shown in Table 1.

**Table 1:** Patients characteristics

Characteristic	EPI 120			EPI 120 + E			EPI 40 + EP0		
	No.		%	No.		%	No.		%
Median age, years		55			54			57	
Range		29-70			33-68			32-70	
TNM									
T2	56		86.2	44		80.0	52		88.1
T3-4	9		13.8	11		20.0	7		11.9
N0	43		66.2	36		65.5	41		69.5
N1	22		33.8	19		34.5	18		30.5
Primary histology									
Ductal carcinoma	58		86.6	46		83.6	45		75.0
Lobular carcinoma	6		9.0	6		10.9	12		20.0
Ductal/lobular carcinoma	2		3.0	2		3.6	3		5.0
Mucoid carcinoma	1		1.5	1		1.8	0		0.0
Grading									
2	16		25.0	22		40.0	13		21.7
3	48		75.0	33		60.0	47		78.3

On first presentation, an incisional biopsy (0.5 to 0.8 cm^3^) was performed. Tumour size and response was assessed by the same specialist, according to the WHO criteria [38] by the clinical measurement of the changes in the product of the two largest diameters recorded in two successive evaluations. Tumour progression (PD) was defined as an increase of at least 25% in tumour size; stable disease (SD) as an increase of less than 25%, or a reduction of less than 50%; partial response (PR) as a tumour shrinkage greater than 50%; and complete response (CR) as the complete disappearance of all clinical signs of disease. Pathologic complete response (pCR) was defined as the absence of neoplastic cells in the breast and in the axillary lymph nodes after histology. Surgery (quadrantectomy or modified radical mastectomy in association with full axillary node dissection) was planned after clinical reassessment. All patients subjected to quadrantectomy underwent irradiation of the residual breast (60 Gy delivered over 6 weeks). All patients received post operatively 6 cycles Cyclophosphamide, Methotrexate and 5-Fluorouracile (CMF regimen). Patients with positive oestrogen receptor primary tumour received tamoxifen (20 mg/daily), irrespective of the trail arm, starting after chemotherapy, up to progression or for a maximum of five years. All the recommended treatment modifications for hematologic toxicities used were previously described [39]. Toxicity was evaluated on the basis of National Cancer Institute Common Toxicity Criteria for Adverse Events, Version 3.0. The study was approved by the local ethics committee. Written informed consent was obtained from all patients before randomization.

### Histopathologic grade and immunohistochemistry

Immunohistochemical evaluation for routine markers (bcl-2, p53, HER2, ER, PgR, and Ki67), as described elsewhere [5, 40], was performed on paraffin-embedded tumour samples of whole tumour sections obtained at diagnosis. The antibodies, sources, and protocols used for the other markers are previously described [41]. Immunohistochemistry for all markers was performed on 5μ sections of tissue microarray containing two 1-mm diameter cores taken from selected morphologically representative tumour regions from the incisional biopsy (Table 2). Quality control was assessed on each block by haematoxylin and eosin staining. The Envision HRP kit (Dako; Cambridgeshire, United Kingdom) system was used for subsequent visualization. Staining was assessed in the nucleus for HIF1 α, Ki67, ER, PgR and p53, nucleus and cytoplasm for PHD1, PHD2, PHD3, TP, EPO, and EPO-R; membrane for CAIX, and HER2; cytoplasm for GLUT-1, Bcl-2 and VEGF. All sections had a negative control slide (no primary antibody) of an adjacent section to preclude nonspecific staining. Positive controls included breast carcinomas known to exhibit high levels of each marker. A tumour was considered positive if more than 10% of the surface area of the sample contained tumour cells. All immunohistochemically stained sections were assessed by light microscopy by two pathologists simultaneously at Nuffield Department of Clinical Laboratory Sciences, University of Oxford, UK. The observers were blinded to the patient’s clinical characteristics, patients’ outcome and samples’ origin. Intensity was semi-quantitatively assessed: 0 (no staining), 1 (weak staining), 2 (moderate staining), or 3 (strong staining) for nuclear HIF1-alpha, PHD1, PHD2, PHD3, CAIX, VEGF, TP, GLUT-1, EPO and EPO-R. The cut-off for PgR, ER, HER2, p53, bcl-2, Ki67 was as previously reported [41]. For the quantification of tumour angiogenesis CD31, a previously reported 25-point Chalkley eyepiece graticuleas [42], was used to count vascular spots identified by scanning the tumour at 40-100 by two observers over a conference microscope. Microvessels were defined as any immunoreactive endothelial cell (EC)s separate from adjacent microvessels. Vessels within the sclerotic body of the tumour were not included. Counting at x250 magnification was then performed by rotating the graticule in the eyepiece to where the maximum number of dots overlay stained vessels. The mean of three counts was used in the subsequent analysis and tumours with counts >7 were considered high vascularity. Tumours with counts of less than 7 were considered as low (0-4) and median (5-6) vascularity. Some of the markers scores were missing due to insufficient tumour or unsatisfactory staining. The approach to missing cases was to exclude cases where the value of the covariate under study was missing in the univariate analysis and to exclude cases when 1 or more covariates were missing in the multivariate analysis.

**Table 2:** Biological markers considered in this study

IHC Molecular Markers	Localization	Median	Mean	95% CI
Tissue microarray				
VEGF Intensity	Cytoplasm	1.0	0.9	0.8 to 1.0
HIF-1a Intensity	Nucleus	0.0	0.5	0.4 to 0.6
CA9 Intensity	Membrane	0.0	0.4	0.3 to 0.6
TP Intensity	Cytoplasm	0.0	0.5	0.4 to 0.7
TP Intensity	Nucleus	1.0	1.1	0.9 to 1.3
GLUT-1 Intensity	Cytoplasm	1.0	1.2	1.0 to 1.4
PHD2 Intensity	Cytoplasm	1.0	1.2	1.0 to 1.4
PHD2 Intensity	Nucleus	0.0	0.5	0.4 to 0.6
EPO Intensity	Nucleus	2.0	2.0	1.9 to 2.2
EPO Intensity	Cytoplasm	2.0	2.1	2.0 to 2.3
EPO-R Intensity	Nucleus	3.0	2.2	2.1 to 2.4
EPO-R Intensity	Cytoplasm	3.0	2.8	2.7 to 2.9
CD 31		LOW (79)	MED (40)	HIGH (45)
**IHC Routine markers**		**Median**	**Mean**	**95% CI**
ER	Nucleus	1.0	0.8	0.7 to 0.9
PgR	Nucleus	1.0	0.5	0.4 to 0.6
Bcl2	Cytoplasm	1.0	0.8	0.7 to 0.9
HER2	Membrane	0.0	0.2	0.1 to 0.2
p53	Nucleus	1.0	0.6	0.5 to 0.6
**Blood Routine Markers**		**Median**	**Mean**	**95% CI**
HB*	Blood	13.6	13.6	13.4 to 13.7
HCT*	Blood	41.0	41.0	40.5 to 41.4

Note: Markers considered in the analysis had < 20% of missing values (ie the marker was assessed in at least or more than 80% of the patients) or < 20% positivity in sample staining (ie, the marker stained positive in at least 20% of the samples).

Abbreviations: HIF-1 alpha, hypoxia-inducible factor 1 alpha; TP, tymidilato phosphorilasy;GLUT-1; PHD2; EPO; CA9; VEGF, vascular endothelial growth factor; ER, estrogen receptor; PgR, progesterone receptor. Haemoglobin (HB), Hematocritus (HCT).

### Statistical methodology

The methodology adopted for this study has been previously described in Generali et al. [41, 43]. Here, we provide a brief description for convenience. For data analysis we used a particular regression approach called *elastic net* (see Zou and Hastie [44]), which addresses the problem of variable selection and regularization in multivariate analyses by using least-square penalization. More specifically, the elastic net approach performs least-square minimization while enforcing a constraint on a combination of the sum of the absolute values of the regression coefficients and the sum of their squares. This constraint enables efficient variable selection and encourages a grouping effect, where strongly correlated predictors tend to stay in or out of the model together.

The markers introduced in the model are described in Table 2, and median, mean, and 95% quintiles are provided. The categorization of the markers was based on the variables as described earlier. The clinical variables introduced in the model were tumour size (0 if T ≤ 2, 1 if T > 2), nodal status (negative vs. positive), age, tumour grade, histological type, and treatment. All scores and clinical variables were standardized before being introduced in the model by applying location and scale transformation as suggested in Zou and Hastie [44].

The approach used to missing data was pairwise deletion (also known as available-case analysis) meaning that each analysis used all cases with no missing values for the variables involved in the analysis. This approach guarantees that the results are unbiased if the ‘missing completely at random assumption’ is satisfied [45], which can be considered as tenable in the study presented here. An alternative to the missing data approach adopted here is multiple imputation using chained equations [46]. Despite its popularity mainly due to the extreme flexibility it offers, this methodology still lacks a general theoretical justification, hence it was not considered for the analysis presented in this work.

Finally, an iterative leave-one-out approach was used to test the model. More specifically, at each iterations, one case (i.e. a single patient) was left out from the analysis, a fit of the model was produced for the remaining cases and a treatment response prediction was made for the left out case. This allowed testing of the models’ ability to predict treatment response, specificity and sensitivity, and respective area under the receiver operator characteristic curve, were estimated using these predictions.

All the analyses presented were performed using the R software (http://cran.r-project.org) using the packages elastic-net and glm-net.

## SUPPLEMENTARY MATERIALS FIGURES AND TABLE


