# Supplementary material for: Hypoxia-related biological markers as predictors of epirubicin-based treatment responsiveness and resistance in locally advanced breast cancer
Source: Oncotarget. 2017 Aug 14;8(45):78870–81. doi: 10.18632/oncotarget.20239 (PMC5668004; doi:10.18632/oncotarget.20239)
Supplement: Supplementary file 1 [file oncotarget-08-78870-s001.pdf]

Hypoxia-related biological markers as predictors of epirubicin-based treatment responsiveness and resistance in locally advanced breast cancer

SUPPLEMENTARY MATERIALS

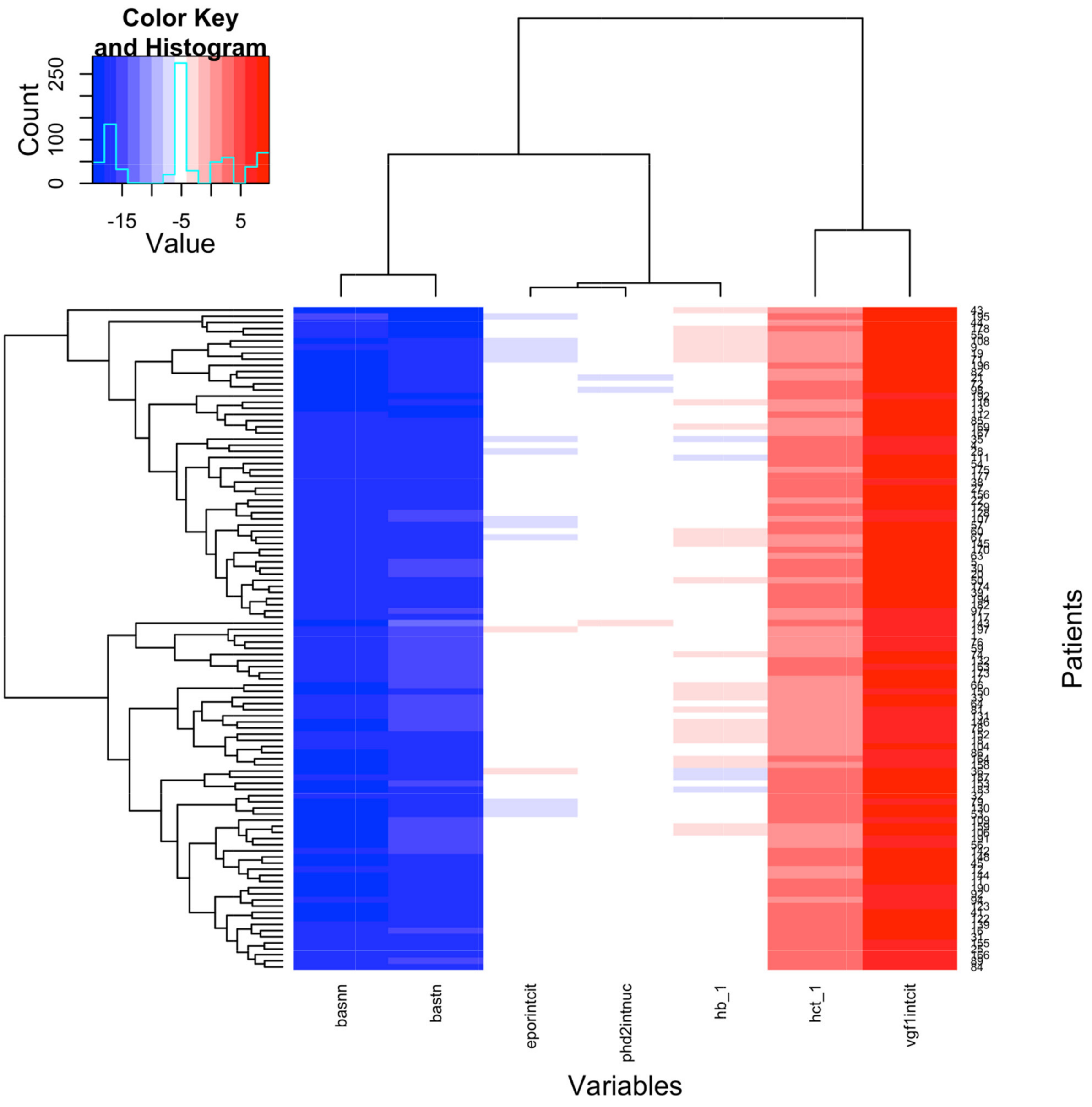

Supplementary Figure 1: Factors Associated With Disease Free Survival.

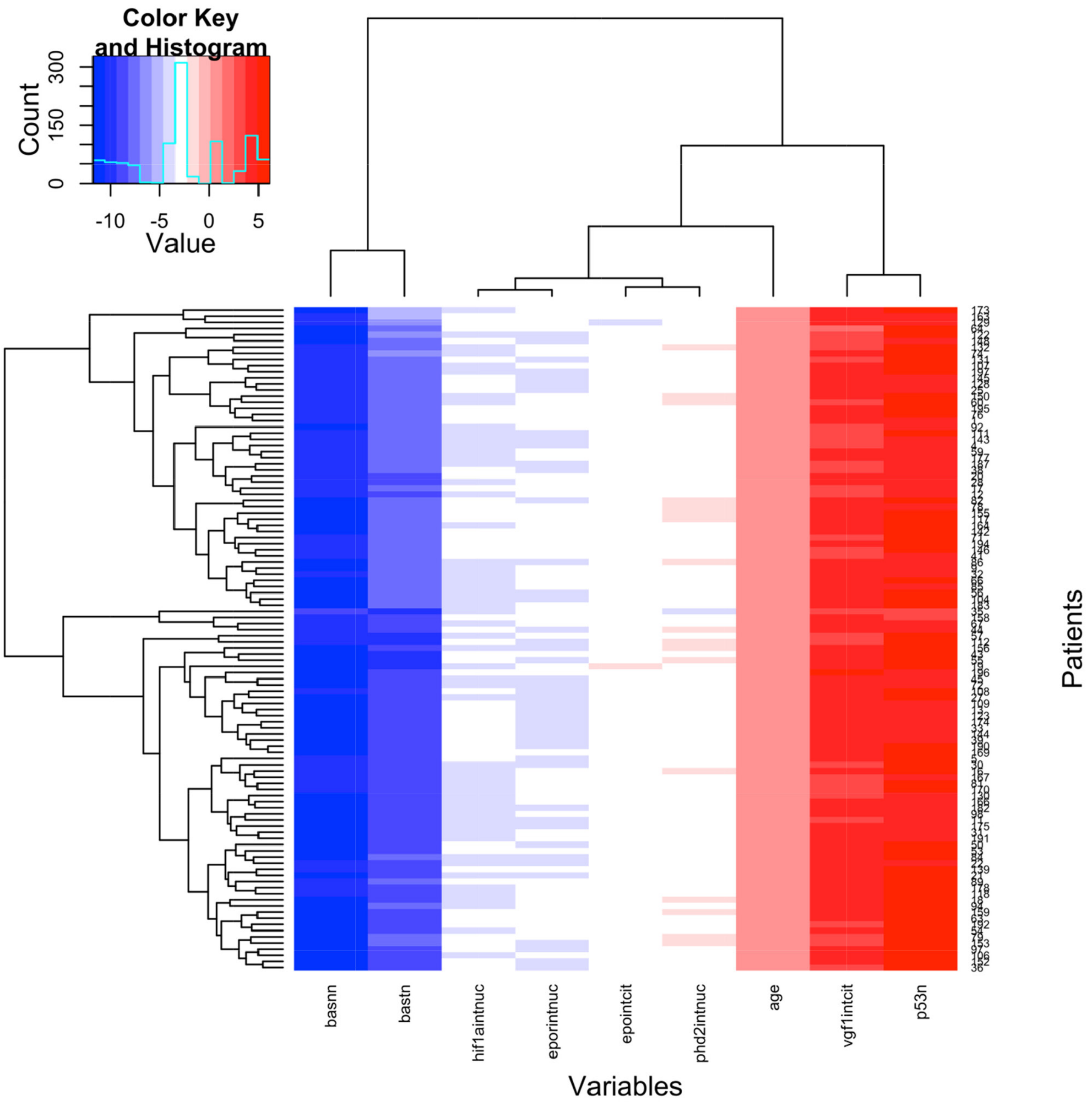

Supplementary Figure 2: Factors Associated With Overall Survival.

Supplementary Table 1: Treatment activity

Response	EPI 120		EPI 120 + EPO		EPI 40 + EPO	
	No. 63	%	No. 55	%	No. 58	%
Progression	0	0	0	0	3	5.2
Stable disease	6	9.5	1	1.8	3	5.2
Partial Response	41	65.1	36	65.5	26	44.9
Complete Response	16	25.4	18	32.7	26	44.8
Overall Response Rate	57	90.5	54	98.2	52	89.7
